# Graphene Oxide Induced Surface Modification for Functional Separators in Lithium Secondary Batteries

**DOI:** 10.1038/s41598-019-39237-8

**Published:** 2019-02-21

**Authors:** Ju Young Kim, Dong Ok Shin, Kwang Man Kim, Jimin Oh, Jumi Kim, Seok Hun Kang, Myeong Ju Lee, Young-Gi Lee

**Affiliations:** Research Group of Multidisciplinary Sensors, Electronics and Telecommunications Research Institute (ETRI), Daejeon, 34129 Republic of Korea

## Abstract

Functional separators, which have additional functions apart from the ionic conduction and electronic insulation of conventional separators, are highly in demand to realize the development of advanced lithium ion secondary batteries with high safety, high power density, and so on. Their fabrication is simply performed by additional deposition of diverse functional materials on conventional separators. However, the hydrophobic wetting nature of conventional separators induces the polarity-dependent wetting feature of slurries. Thus, an eco-friendly coating process of water-based slurry that is highly polar is hard to realize, which restricts the use of various functional materials dispersible in the polar solvent. This paper presents a surface modification of conventional separators that uses a solution-based coating of graphene oxide with a hydrophilic group. The simple method enables the large-scale tuning of surface wetting properties by altering the morphology and the surface polarity of conventional separators, without significant degradation of lithium ion transport. On the surface modified separator, superior wetting properties are realized and a functional separator, applicable to lithium metal secondary batteries, is demonstrated as an example. We believe that this simple surface modification using graphene oxide contributes to successful fabrication of various functional separators that are suitable for advanced secondary batteries.

## Introduction

Until now, lithium ion batteries have been widely used as excellent energy storage devices owing to their high energy density and reasonable power density^1–4^. It is predicted that this superior energy storage device will extend its usefulness to energy storage systems, a power source for the Internet of Things and implantable medical devices^5,6^. To this end, components constituting lithium secondary batteries should be developed to exhibit advanced functions. The separator is one of the essential components that allows easy ionic transport and prevents electronic conduction^7–10^. The separator used in lithium ion batteries is generally a polymeric membrane with micrometer-scale porosity, but many recently proposed functional separators have additional features: high thermal stability, high rate operation, lithium dendrite suppression, flame-retardation and so on^11–21^. For this purpose, the polymer scaffold was modified to form a multiscale or ordered porosity, and the hybrid composite separators with functional inorganic nanomaterials were demonstrated. Also, the separator consisted of functional polymers with a core-shell structure or a polarity-controlled surface. Among them, the common and facile method is the additional deposition of functional materials on conventional separators.

In this regard, surface wetting properties of separators are highly important because the uniform deposition process of new, functional materials is significantly dependent on the interfacial adhesion or wettability of separators^22,23^. However, conventional polymeric separators consist of polyolefin fiber structure that exhibit hydrophobic behavior. On this separator, the polar slurries might experience a dewetting problem, which makes a uniform coating of functional materials difficult. Thus, less polar solvents such as n-methyl-2-pyrrolidone which enables moderate wetting with hydrophobic conventional separators have usually been used to form a slurry containing functional materials and polymeric binders, but the toxicity and high cost of organic solvents are fundamental issues for the development of cost-effective lithium ion batteries through eco-friendly processes^22^. In addition, the use of functional nanomaterials dispersible in a polar solvent is strictly limited. To overcome these problems, significant research has been performed that includes mussel-inspired dopamine coating of separators^22,24–26^.

This paper presents a facile method to tune surface properties of conventional separators by solution-based, rapid coating of graphene oxide (GO). Through dip-coating of conventional separators in a GO solution with a polarity designed for uniform wetting, the separator surface can be completely covered by GO flakes. Thus, the hydrophilic wetting nature of the modified separator can be realized because of many hydrophilic functional groups existing on the GO surface and the morphological change of the GO covered separator. The resulting properties provide superior wettability of eco-friendly water-based slurry and corresponding uniform deposition of functional materials. As an example, we fabricated a GO-SiO_2_ composite layer coated separator to demonstrate its usefulness in lithium metal secondary batteries. The superior cycling performance of the cell with the GO-SiO_2_ coated separator was achieved compared to the cell with pristine separator.

### Experimental procedures

The GO was synthesized from nature graphite (SP1 Bay Carbon) by the modified Hummers method^27,28^. For uniform coating of GO flakes on the separator surface, ~0.02 wt% GO dispersion in a solvent mixture of isopropyl alcohol (IPA)/water (20/1 by volume ratio) was prepared. Prior to GO coating, sonication for 30 min was performed for stable dispersion. A conventional separator (Asahi Kasei) was dipped in the GO solution for 15–30 s, and after that, the wet separator was dried for several min in ambient conditions. For deposition of the GO-SiO_2_ composite layer on the separators, ~1 wt% GO aqueous solution with SiO_2_ nanoparticles (Sigma-Aldrich) and sodium carboxymethyl cellulose (CMC) binder (~1 wt% GO:SiO_2_:CMC = 100:3:0.08 by weight) was used after strong stirring. The thickness-controlled doctor blade method was performed to form the composite film on the separators. The GO-SiO_2_ coated separator was annealed at 60 °C in a vacuum over 6 h to remove any traces of water. For cell fabrication, we used a commercially available LiCoO_2_ (Umicore, D_50_ = 10 μm) cathode, a liquid electrolyte (EnChem Co. Ltd.) of 1 M LiPF_6_ in ethylene carbonate (EC)/diethyl carbonate (DEC) with 2 wt% vinylene carbonate (VC) additive and lithium metal (Honjo Corporation) with ~300 μm thickness.

Nanoscale morphologies were characterized by scanning electron microscopy (SEM, Hitachi S-4800). The water contact angle on the pristine or modified separators was measured with Surface Electro Optics (PNX 150). Ionic conductivity was determined from complex impedance spectra measured using a frequency response analyzer (Solartron HF 1225 Gain-Phase Analyzer) in a frequency range of 10^−1^-10^5^ Hz. Electrochemical performance of lithium metal secondary batteries was investigated using a cycle tester (Toyo System).

## Results and Discussion

**Figure 1:**
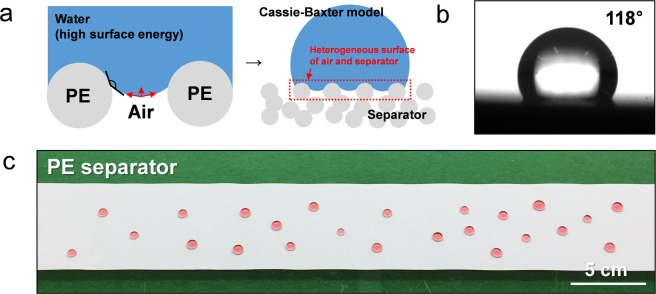
(**a**) Schematic illustration of a solid-liquid-vapor interface of the conventional hydrophobic separator structure for water. (**d**) Water contact angle measurement and (**c**) photograph of large-area wetting feature on the conventional hydrophobic separator. For visualization, Co(NO_3_)_2_·6H_2_O dissolved water was used.

In general, the polyolefin separators exhibit hydrophobic wetting nature due to i) the surface polarity and ii) the porous structural factor^29,30^. Specifically, the low chemical polarity between carbon and hydrogen in polyolefin leads to hydrophobicity, which is intensified by the re-entrant curvature of the separator with a tangled fiber shape^29^. Figure 1a schematically describes a solid-liquid-vapor interface of a hydrophobic separator structure for water, where the net traction of the liquid-vapor interface is upward owing to a high water contact angle on the polyolefin surface. Such a net force prevents water from penetrating into the separator, which forms a heterogeneous surface consisting of air and a hydrophobic separator. This phenomenon can be interpreted as a Cassie-Baxter model^30^. As a result, this porous olefin separator shows a hydrophobic dewetting nature with a water contact angle of ~118° (Fig. 1b,c), and it is anticipated to be hard for an aqueous slurry solution to be uniformly coated^22^.

**Figure 2:**
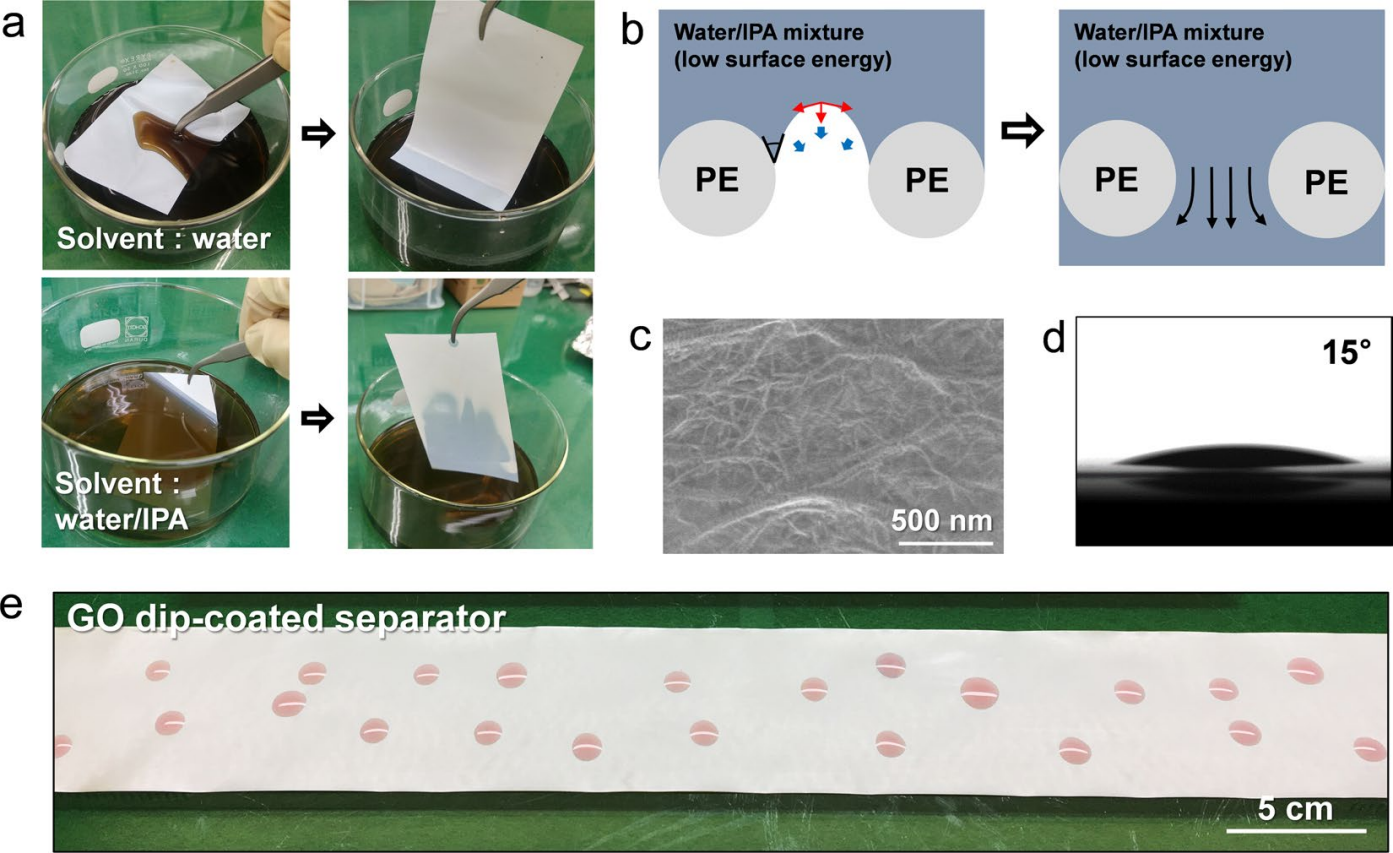
(**a**) Photographs of graphene oxide (GO) dip-coating depending on the polarity of GO solution. (**b**) Schematic illustration of wettability of the solvent with low surface energy on the conventional hydrophobic separator. (**c**) SEM image of the GO dip-coated separator. (**d**) Water contact angle measurement and (**e**) photograph of large-area wetting feature on the GO dip-coated separator. For visualization, Co(NO_3_)_2_·6H_2_O dissolved water was used.

Therefore, a surface treatment to change the hydrophobic surface nature of the separators to hydrophilic should be performed for the uniform and scalable coating of aqueous slurry solution. To this end, a facile dip-coating of the separators in GO solution was designed (Fig. S1). GO, used widely for diverse chemical and optoelectric applications, is an intriguing two-dimensional nanomaterial in virtue of its mass producibility, solution-processability and controllable defects on a basal plane^27,31,32^. In this proposed process, we anticipated that several layers of flexible GO flakes with a hydrophilic surface via oxygen functional groups would physically and conformally adhere to the separator surface through the dip-coating process, utilizing the size difference between the GO (several micrometer scale) and separator pores (hundreds nanometer scale)^28^. However, the above-mentioned dewetting feature of conventional separators for aqueous solutions disturbed the surface modification of the separator (Figs 2a and S2). Nevertheless, a simple solvent change from water, with a high surface energy (72.80 mJ m^−2^), to one with a low surface energy can solve the wetting issue. As shown in Fig. 2b, the solvent with a low surface energy shows a relatively low contact angle on the olefin surface, and thus the net traction of the liquid-vapor interface in this situation can be directed downward. This phenomenon leads to failing of the heterogeneous layer containing air pockets and favorable wetting of the solvent with the separator. To decrease the surface energy of the GO aqueous solution, the isopropyl alcohol (IPA) with a relatively weak intermolecular interaction (23.0 mJ m^−2^) was added, and the stable dispersion of GO in a water/IPA mixture could be obtained owing to the high dispersibility of GO in diverse solvents (Fig. S3)^33^. Eventually, when the GO dispersion in the water/IPA mixture was used for surface coating of the separators, the evaporation-induced deposition of GO on the separators was successfully realized within several minutes, in contrast to the use of aqueous GO solutions (Figs 2a, S2 and S4). The SEM image confirmed the conformal deposition of GO thin enough that the separator structure could be observed on opposite side (Fig. 2c)^34^. Notably, this GO-induced surface modification enables a distinct transition of wetting properties, modifying the surface chemistry and the re-entrant structure of the pristine separators^28,29^. As shown in Fig. 2d,e, the water contact angle on this modified separator significantly decreases, showing a hydrophilic nature (water contact angle: ~15°) on the large area.

**Figure 3:**
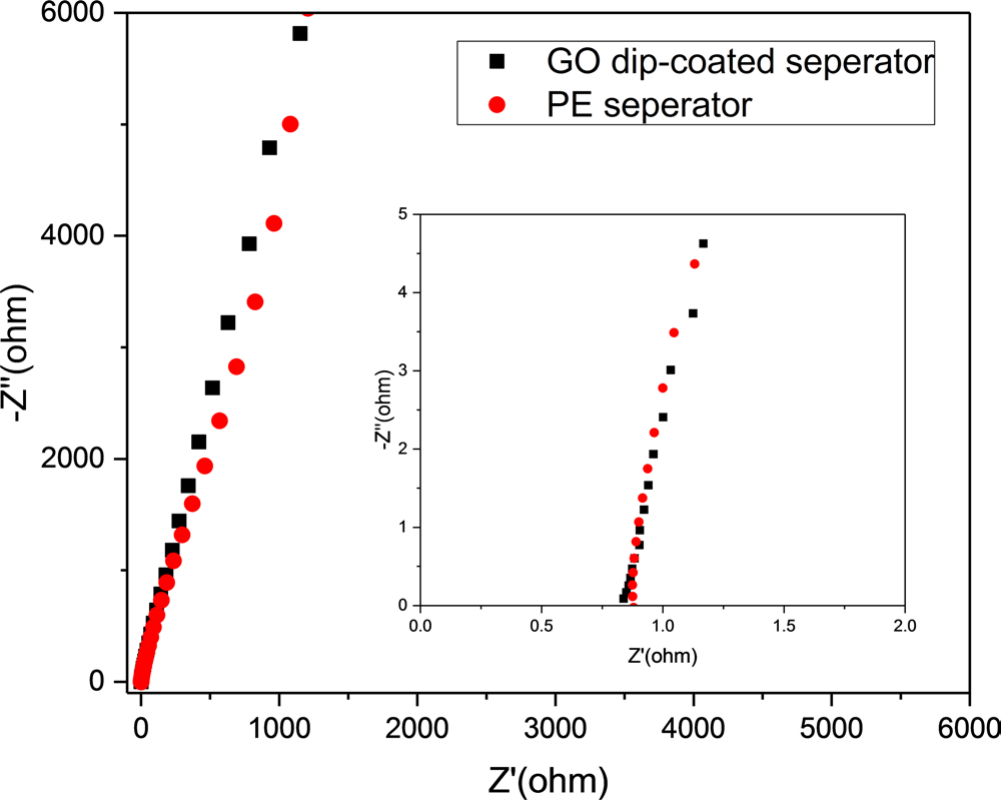
The Nyquist plot of 1 M LiPF_6_ in EC/DMC using the GO dip-coated separator and the conventional separator, respectively.

One of the remarkable results is lithium ion transport within this surface modified separator. In spite of the physical blocking of GO as shown in the SEM image of Fig. 2c, the GO dip-coated separator exhibits ionic conductivity (0.596 mS cm^−1^) similar to the pristine separator (0.581 mS cm^−1^) (Fig. 3). We inferred that this was owing to i) the smooth ion transport through numerous defect sites on the basal plane of the GO and ii) the ultrathin deposition of GO (a few nanometers) compared with the total separator thickness (tens of micrometer scale)^35,36^. Specifically, the GO seems to macroscopically block the ion transport, but in microscopic scale, many defects or functional groups on the GO can help ionic conduction due to having an appropriate size for lithium ions to pass^35,36^. Moreover, the deposited GO is significantly thin compared to the total thickness of the separator, and thus even if there is any retardation in lithium transport, the negative effect on the ionic conduction due to the GO layer can be minimized. These results strongly suggest that this surface modification through thin GO coating can improve the surface wettability without significant degradation of lithium ion transport, which is highly important to the operation of lithium ion batteries.

**Figure 4:**
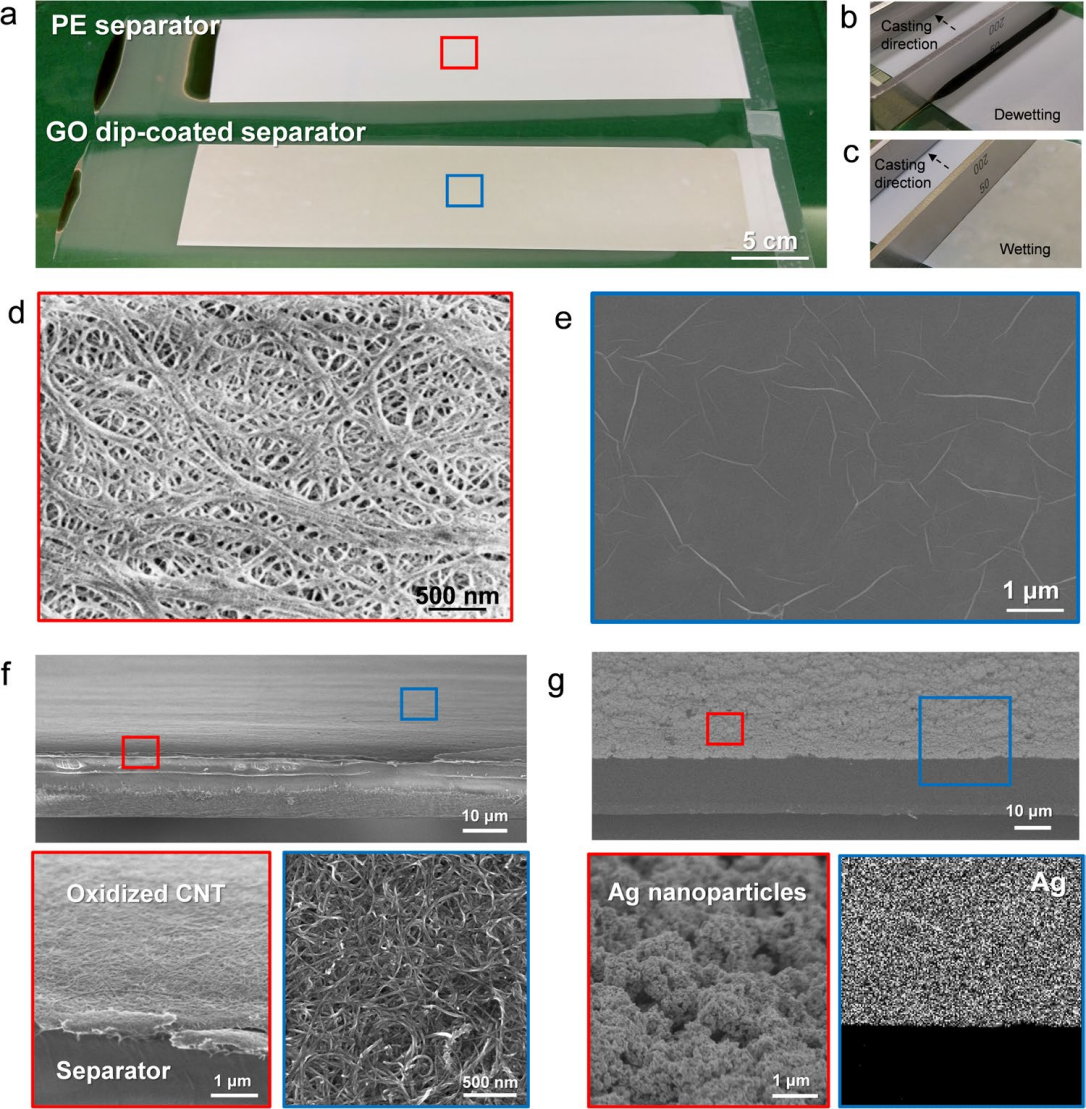
Photograph of (**a**) GO coating depending on the separators, (**b**) dewetted GO solution on the conventional separator and (**c**) completely wetted GO solution on the surface modified separator during the doctor blade casting. SEM images of (**d**) red marked region and (**e**) blue marked region in (**a**). SEM and EDX images of (**f**) oxidized carbon nanotube, (**g**) Ag nanoparticles coated separator.

On this surface modified separator, the facile coating of water-based slurry with diverse functional materials can be realized. As a model system of water-based slurries, the GO aqueous solution with a concentration of ~1 wt% was prepared. As shown in Fig. 4a,b, the high polarity of the GO aqueous solution prevents the uniform coating of GO flakes on the pristine separator, but owing to the enhanced wettability on the surface modified separator, the water-based slurry containing GO flakes can be uniformly coated, which is confirmed via large-scale demonstration (Fig. 4a,c). In addition, the SEM measurement was performed to investigate the uniform GO coating at the microscopic level and the densely GO covered morphology was observed (Fig. 4e). It shows a distinct contrast to one of pristine separator, where it is hard to observe the GO flakes (Fig. 4d). In addition, oxidized carbon nanotube and Ag nanoparticles coated separators were demonstrated using similar aqueous slurries, which exhibit the uniform coating feature (Fig. 4f–g). From these results, it is noted that this surface modified separator can be utilized for the uniform deposition of diverse functional materials, regardless of the polarity of slurries.

**Figure 5:**
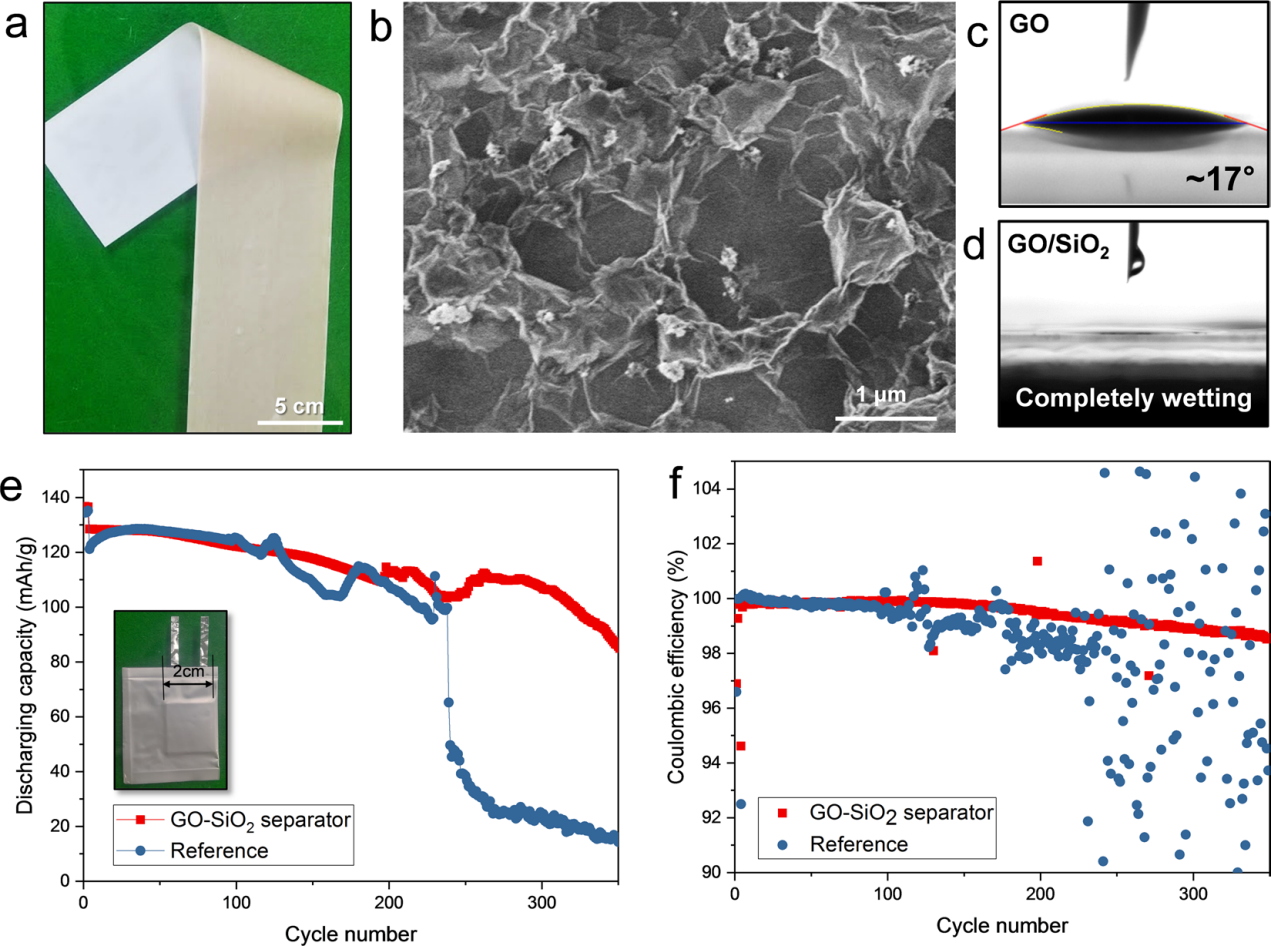
(**a**) Photograph and (**b**) SEM image of the GO-SiO_2_ coated separator. Water contact angle measurement of (**e**) GO coated separator and (**d**) GO-SiO_2_ coated separator. (**e**) Cycling performance and (**f**) Coulombic efficiency of lithium metal secondary batteries using the GO-SiO_2_ coated separator.

To check the electrochemical performance of secondary batteries that have a functional separator, the GO-SiO_2_ composite layer coated separator was fabricated for lithium metal secondary batteries. The well-mixed, water-based slurry containing GO and SiO_2_ nanoparticles was prepared and the uniform deposition with <5 μm thickness was successfully realized on the surface modified separator (Figs 5a and S5). Lithium metal secondary batteries utilizing a lithium metal anode instead of the conventional graphite anode, aim to achieve an ultrahigh energy density storage device that uses the lowest electrochemical potential and high specific capacity of lithium, but it suffers from the uncontrolled, undesired lithium dendrite growth^37–43^. This structural phenomenon can be alleviated to some extent by a mechanically strong blocking layer. Thus, a functional separator coated with GO capable of in-plane mechanical robustness was presented^44–48^. In addition, in order to obtain additional life of lithium metal, SiO_2_ nanoparticles were incorporated within the GO layer. It is anticipated that lithium dendrites will meet SiO_2_ nanoparticles during repeated cycling, which induces a solid-state conversion reaction^13^. Thus, this electrochemical reaction might prevent further growth of lithium dendrites because the sharp lithium dendrites become a smooth and round shaped lithium alloy, which is an electrically less active structure by lightning rod theory^13,37^. Also, SiO_2_ nanoparticles can prevent strong GO stacking which deteriorate the ionic conductivity owing to tortuous ionic pathway of multilayered structure. As shown in Fig. S6, the XRD results display that the degree of GO stacking decreases as the SiO_2_ nanoparticles are added and corresponding impedance results show that the ionic conductivity of the GO-SiO_2_ coated separator is enhanced compared to one of the GO coated separator.

The SEM image of Fig. 5b shows the composite layer consisting of GO and SiO_2_ nanoparticles on the surface modified separator. In contrast to the separator coated by GO layers (Fig. 4e), the rough GO surface was observed at a microscopic level, owing to the incorporation of SiO_2_ nanoparticles. This roughness induces the enhanced solvent wettability according to Wenzel model (Fig. 5c,d)^49^, which makes a liquid electrolyte uniformly distributed on the lithium metal. This phenomenon generally contributes to the uniform lithium deposition with fewer dendrites^50,51^. For evaluation of lithium metal secondary batteries using this functional separator, the 2 × 2 cm^2^ pouch type full-cell was assembled with a LiCoO_2_ cathode, ~300 μm thick lithium metal, and liquid electrolyte (1 M LiPF_6_ in EC/DEC + 2 wt% VC additive). As shown in Fig. 5c,d, the enhancement of cycling performance at the rate of 1 C was confirmed by using the GO-SiO_2_ coated separator. The cell with the GO-SiO_2_ coated separator exhibited stable operation for ~200 cycles and some fluctuation after that, but the Coulombic efficiency was quite stable up to 350 cycles. Meanwhile, the reference cell using the conventional separator showed severe fluctuation of discharging capacity and Coulombic efficiency after 100 cycles, and cell failure was observed after ~220 cycles. It is clear that the composite layer of GO and SiO_2_ nanoparticles can efficiently prevent the lithium dendrite growth through the physical robustness of GO, the electrochemical conversion reaction of SiO_2_ nanoparticles and the uniform distribution of lithium ions.

## Conclusions

In summary, a surface modification of separators was presented via a simple solution-based coating of GO with many hydrophilic groups for fabrication of functional separators. This strategy was easily performed within several minutes and dramatically changed the wetting nature from hydrophobic to hydrophilic, which was mainly achieved by modifying the surface polarity and the porous structure of the separators. Our findings indicate that slurries with many functional materials can be coated on this separator surface, regardless of the polarity of the slurries. In addition, this surface modification approach preserved the ionic conduction through defect-induced ion transport and thin deposition thickness. As a model system, deposition of a GO-SiO_2_ composite layer was demonstrated on the proposed surface modified separator, and the enhanced cycling performance of the GO-SiO_2_ coated separator was confirmed in lithium metal secondary batteries. We believe that this facile surface modification of the separator via GO coating will contribute to fabricating various functional separators that incorporate intriguing nanomaterials, such as emerging 1- or 2-dimensional materials (carbon nanotube, graphene, MoS_2_, WS_2_, Mxenes) and novel ceramic materials, which will achieve enhanced performance in the membrane field as well as for advanced lithium secondary batteries.

## Supplementary information

Supplementary info

## References

[1] Goodenough JB, Kim Y (2010). Challenges for Rechargeable Li Batteriaes. Chem. Mater..

[2] Tarascon JM, Armand M (2001). Issues and challenges facing rechargeable lithium batteries. Nature.

[3] Abraham KM (1993). Directions in secondary lithium battery research and development. Electrochim. Acta.

[4] Scrosati B (2000). Recent advances in lithium ion battery materials. Electrochim. Acta.

[5] Zhang F (2017). 3D printing technologies for electrochemical energy storage. Nano Energy.

[6] Ortiz GF (2009). TiO2 nanotubes manufactured by anodization of Ti thin films for on-chip Li-ion 2D microbatteries. Electrochim. Acta.

[7] Lee H, Yanilmaz M, Toprakci O, Fu K, Zhang X (2014). A review of recent developments in membrane separators for rechargeable lithium-ion batteries. Energy Environ. Sci..

[8] Deimede V, Elmasides C (2015). Separators for Lithium-Ion Batteries: A Review on the Production Processes and Recent Developments. Energy Technology.

[9] Degen I, Peter J, Setauket S (1985). Battery Separators. Chem. Rev..

[10] Deng N (2016). A review on separators for lithium-sulfur battery: Progress and prospects. J. Power Sources.

[11] Kim S (2017). A Flame-Retardant Composite Polymer Electrolyte for Lithium-Ion Polymer Batteries. Electrochim. Acta.

[12] Yoo S (2015). Hierarchical multiscale hyperporous block copolymer membranes via tunable dual-phase separation. Sci. Adv..

[13] Liu K (2016). Extending the Life of Lithium-Based Rechargeable Batteries by Reaction of Lithium Dendrites with a Novel Silica Nanoparticle Sandwiched Separator. Adv. Mater..

[14] Lee JR, Won JH, Kim JH, Kim KJ, Lee SY (2012). Evaporation-induced self-assembled silica colloidal particle-assisted nanoporous structural evolution of poly(ethylene terephthalate) nonwoven composite separators for high-safety/high-rate lithium-ion batteries. J. Power Sources.

[15] Jung Y-C (2015). Ceramic separators based on Li+–conducting inorganic electrolyte for high-performance lithium-ion batteries with enhanced safety. J. Power Sources.

[16] Choi Y, Kim JI, Moon J, Jeong J, Park JH (2018). Electron beam induced strong organic/inorganic grafting for thermally stable lithium-ion battery separators. Appl. Surf. Sci..

[17] Wu H, Zhuo D, Kong D, Cui Y (2014). Improving battery safety by early detection of internal shorting with a bifunctional separator. Nat. Commun..

[18] Zhang J (2014). Sustainable, heat-resistant and flame-retardant cellulose-based composite separator for high-performance lithium ion battery. Sci. Rep..

[19] Shi J (2015). Porous membrane with high curvature, three-dimensional heat-resistance skeleton: a new and practical separator candidate for high safety lithium ion battery. Sci. Rep..

[20] Park S (2016). Multicore-shell nanofiber architecture of polyimide/polyvinylidene fluoride blend for thermal and long-term stability of lithium ion battery separator. Sci. Rep..

[21] Huang J-Q, Zhang Q, Wei F (2015). Multi-functional separator/interlayer system for high-stable lithium-sulfur batteries: Progress and prospects. Energy Storage Materials.

[22] Lee H, Jeon H, Gong S, Ryou M-H, Lee YM (2018). A facile method to enhance the uniformity and adhesion properties of water-based ceramic coating layers on hydrophobic polyethylene separators. Appl. Surf. Sci..

[23] Jeon H (2016). Plasma-assisted water-based Al_2_O_3_ ceramic coating for polyethylene-based microporous separators for lithium metal secondary batteries. Electrochim. Acta.

[24] Ryou MH (2012). Excellent Cycle Life of Lithium-Metal Anodes in Lithium-Ion Batteries with Mussel-Inspired Polydopamine-Coated Separators. Adv. Energy Mater..

[25] Ryou MH, Lee YM, Park JK, Choi JW (2011). Mussel-inspired Polydopamine-treated Polyethylene Separators for High-power Li-ion Batteries. Adv. Mater..

[26] Wang Y (2018). Polyethylene separators modified by ultrathin hybrid films enhancing lithium ion transport performance and Li-metal anode stability. Electrochim. Acta.

[27] Padmajan Sasikala S (2018). Graphene oxide liquid crystals: a frontier 2D soft material for graphene-based functional materials. Chem. Soc. Rev..

[28] Kim BH (2010). Surface Energy Modification by Spin-Cast, Large-Area Graphene Film for Block Copolymer Lithography. ACS Nano.

[29] Tuteja A, Choi W, Mabry JM, McKinley GH, Cohen RE (2008). Robust omniphobic surfaces. Proc. Natl. Acad. Sci..

[30] Cassie ABD, Baxter S (1944). Wettability of porous surfaces. *Trans. Faraday*. Society.

[31] Kim JY (2016). 3D Tailored Crumpling of Block-Copolymer Lithography on Chemically Modified Graphene. Adv. Mater..

[32] Chakrabarti MH (2013). Progress in the electrochemical modification of graphene-based materials and their applications. Electrochim. Acta.

[33] Paredes JI, Villar-Rodil S, Martínez-Alonso A, Tascón JMD (2008). Graphene Oxide Dispersions in Organic Solvents. Langmuir.

[34] Koenig SP, Boddeti NG, Dunn ML, Bunch JS (2011). Ultrastrong adhesion of graphene membranes. Nat. Nanotech..

[35] Gómez-Navarro C (2010). Atomic structure of reduced graphene oxide. Nano Lett..

[36] Zhao X, Hayner CM, Kung MC, Kung HH (2011). Flexible holey graphene paper electrodes with enhanced rate capability for energy storage applications. ACS Nano.

[37] Park J (2016). Micro-Patterned Lithium Metal Anodes with Suppressed Dendrite Formation for Post Lithium-IonBatteries. Adv. Mater. Interfaces.

[38] Chen K-H (2017). Dead Lithium: Mass Transport Effects on Voltage, Capacity, and Failure of Lithium Metal Anodes. J. Mater. Chem. A.

[39] Lu D (2015). Failure Mechanism for Fast-Charged Lithium Metal Batteries with Liquid Electrolytes. Adv. Energy Mater..

[40] Lin D, Liu Y, Cui Y (2017). Reviving the Lithium Metal Anode for High-EnergyBatteries. Nat. Nanotech..

[41] Zheng H (2018). A bifunctional electrolyte additive for separator wetting and dendrite suppression in lithium metal batteries. Electrochim. Acta.

[42] Wang Z, Wang X, Sun W, Sun K (2017). Dendrite-Free Lithium Metal Anodes in High Performance Lithium-Sulfur Batteries with Bifunctional Carbon Nanofiber Interlayers. Electrochim. Acta.

[43] Bobnar J (2018). Fluorinated reduced graphene oxide as a protective layer on the metallic lithium for application in the high energy batteries. Sci. Rep..

[44] Akbari A (2016). Large-area graphene-based nanofiltration membranes by shear alignment of discotic nematic liquid crystals of graphene oxide. Nat. Commun..

[45] Shin WK, Kannan AG, Kim DW (2015). Effective Suppression of Dendritic Lithium Growth Using an Ultrathin Coating of Nitrogen and Sulfur Codoped Graphene Nanosheets on Polymer Separator for Lithium Metal Batteries. ACS Appl. Mater. Interfaces.

[46] Yan K (2014). Ultrathin Two Dimensional Atomic Crystals as Stable Interfacial Layer for Improvement of Lithium Metal Anode. Nano Lett..

[47] Huang J-Q (2015). Permselective Graphene Oxide Membrane for Highly Stable and Anti-Self-Discharge Lithium–Sulfur Batteries. ACS Nano.

[48] Yunbo Z (2015). A graphene-oxide-based thin coating on the separator: an efficient barrier towards high-stable lithium–sulfur batteries. 2D Materials.

[49] Wenzel RN (1936). Resistance of Solid Surfaces to Wetting byWater. Industrial & Engineering Chemistry.

[50] Han M, Kim D-W, Kim Y-C (2016). Charged Polymer-Coated Separators by Atmospheric Plasma-Induced Grafting for Lithium-IonBatteries. ACS Appl. Mater. Interfaces.

[51] Liu W, Lin D, Pei A, Cui Y (2016). Stabilizing Lithium Metal Anodes by Uniform Li-Ion Flux Distribution in Nanochannel Confinement. J. Am. Chem. Soc..

