# Supplementary material for: Graphene Oxide Induced Surface Modification for Functional Separators in Lithium Secondary Batteries
Source: Sci Rep. 2019 Feb 21;9:2464. doi: 10.1038/s41598-019-39237-8 (PMC6385286; doi:10.1038/s41598-019-39237-8)
Supplement: Supplementary file 1 — Supplementary info [file 41598_2019_39237_MOESM1_ESM.docx]

**Graphene Oxide Induced Surface Modification for Functional Separators in Lithium Secondary Batteries**

Ju Young Kim^*^, Dong Ok Shin, Kwang Man Kim, Jimin Oh, Jumi Kim, Seok Hun Kang, Myeong Ju Lee, Young-Gi Lee^*^

*Research Group of Multidisciplinary Sensors, Electronics and Telecommunications Research Institute (ETRI), Daejeon 34129, Republic of Korea*

*Corresponding authors.

E-mail address: juyoung@etri.re.kr (J.Y. Kim), lyg@etri.re.kr (Y.-G. Lee).


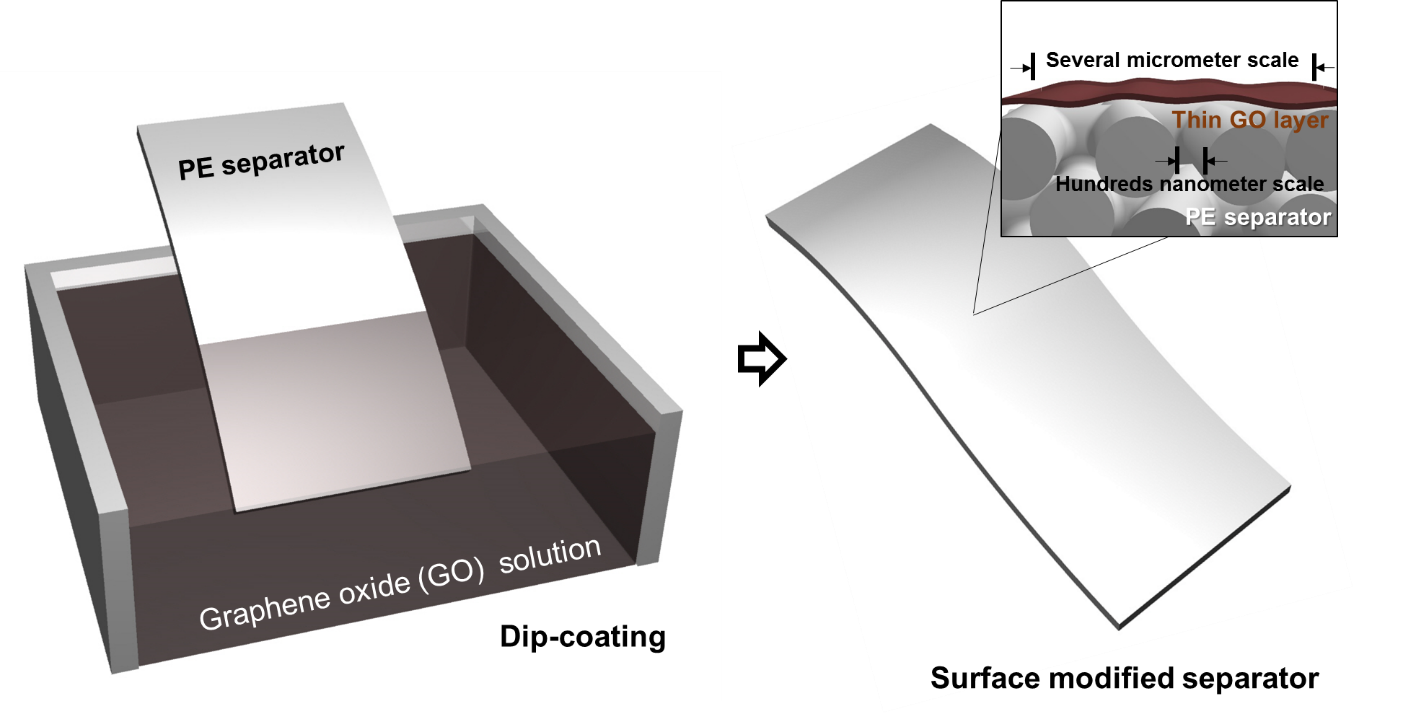


Figure S1. Schematic illustration of the graphene oxide induced surface modification of the conventional separators.


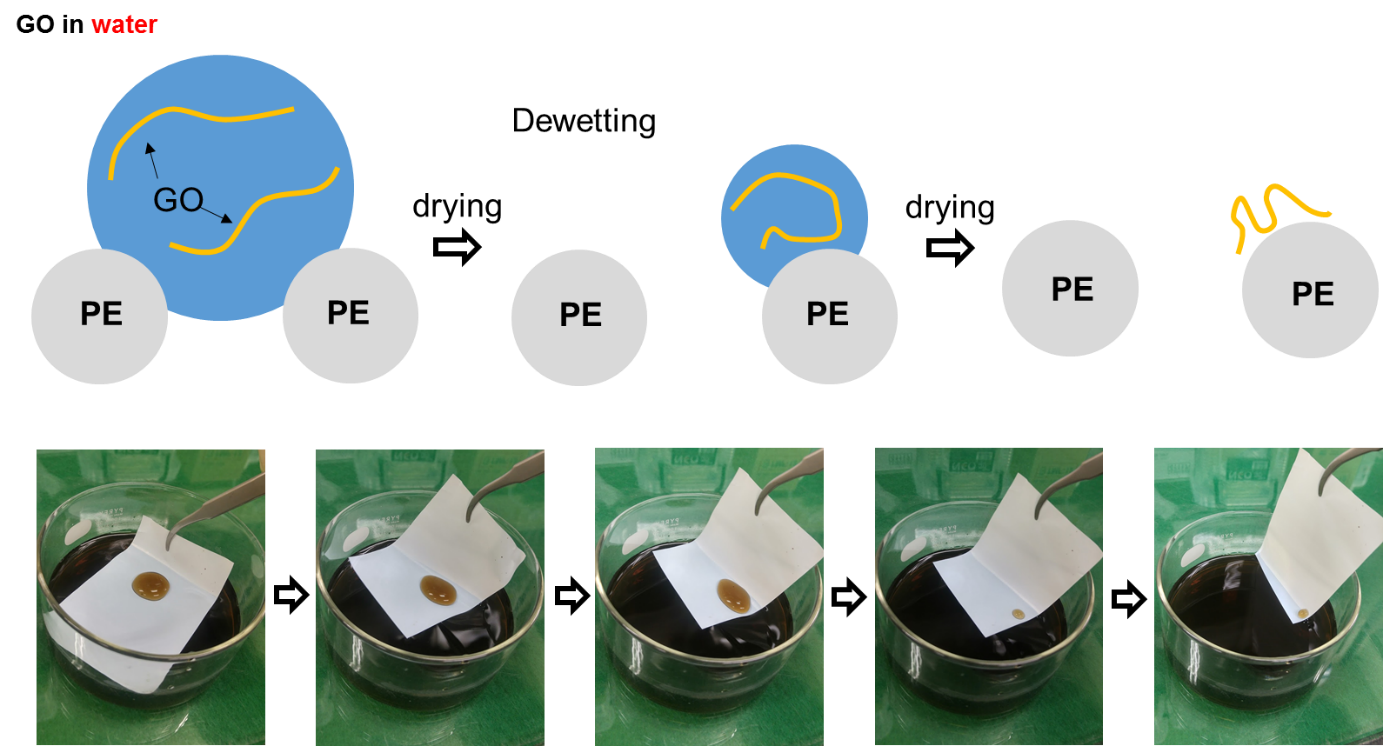


Figure S2. Schematic illustration and photograph of the separator coating by the water-based solution of graphene oxide.


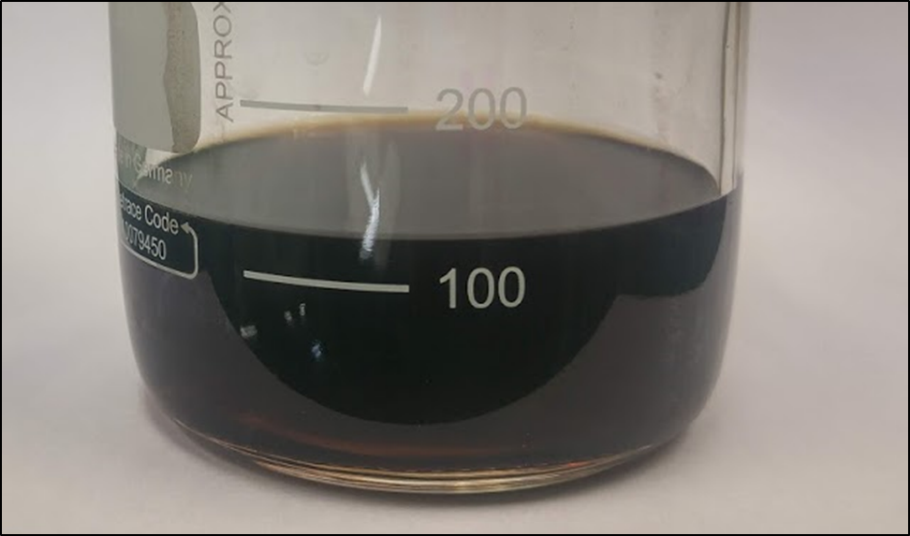


Figure S3. Photograph of the stable GO dispersion in IPA/water solvent.


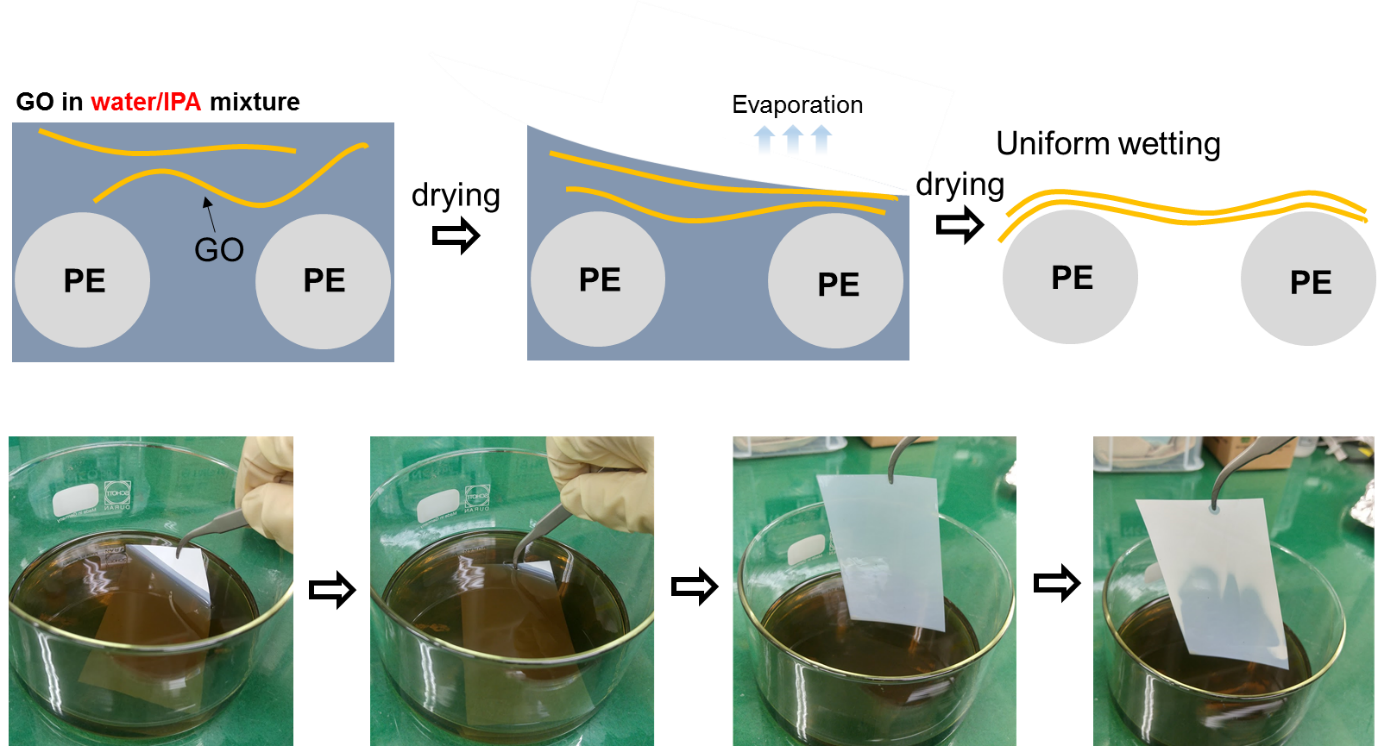


Figure S4. Schematic illustration and photograph of separator coating by the water/IPA (1/ 20 by volume ratio)-based solution of graphene oxide


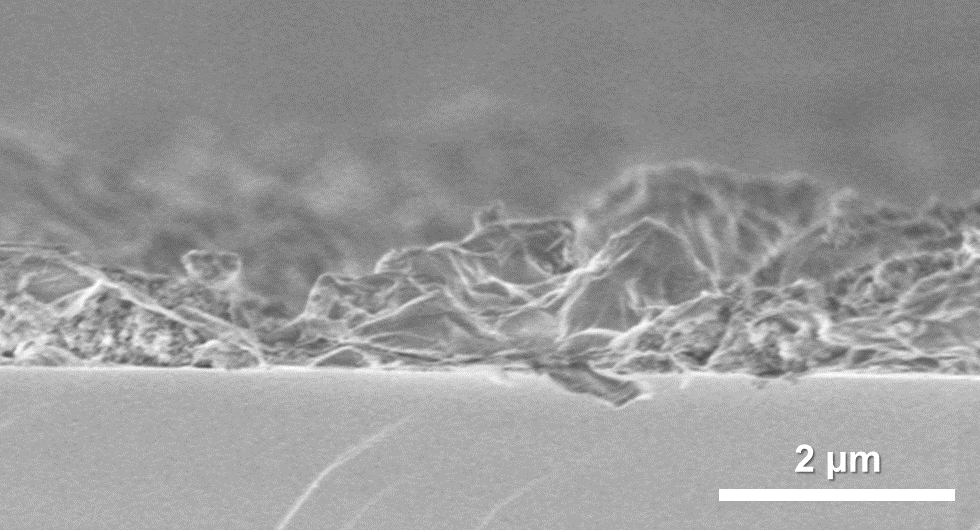


Figure S5. The side view SEM image of GO-SiO_2_ coated separator. For clear observation, the GO-SiO_2_ coating on silicon substrate was performed with same coating process, instead of separator.


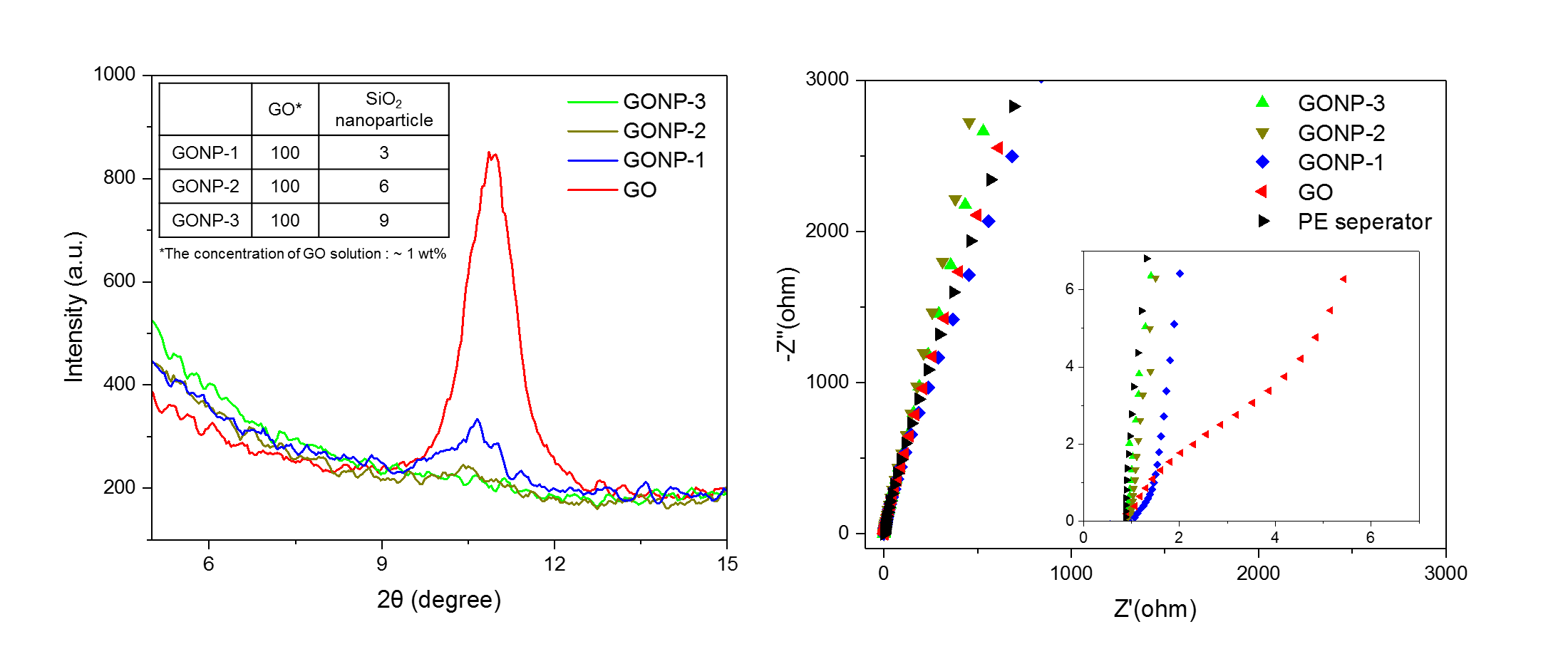


Figure S6. XRD and impedance results of the GO-SiO_2_ separators in relation to the blending ratio of GO and SiO_2_ nanoparticles.
